# Antecedents of hospital admission for deliberate self-harm from a 14-year follow-up study using data-linkage

**DOI:** 10.1186/1471-244X-10-82

**Published:** 2010-10-18

**Authors:** Francis Mitrou, Jennifer Gaudie, David Lawrence, Sven R Silburn, Fiona J Stanley, Stephen R Zubrick

**Affiliations:** 1 Telethon Institute for Child Health Research, Centre for Child Health Research, The University of Western Australia. PO Box 855, West Perth, WA. 6872, Australia; 2 Centre for Developmental Health, Curtin Health Innovation Research Institute, Curtin University of Technology, Perth, Western Australia, Australia

## Abstract

**Background:**

A prior episode of deliberate self-harm (DSH) is one of the strongest predictors of future completed suicide. Identifying antecedents of DSH may inform strategies designed to reduce suicide rates. This study aimed to determine whether individual and socio-ecological factors collected in childhood and adolescence were associated with later hospitalisation for DSH.

**Methods:**

Longitudinal follow-up of a Western Australian population-wide random sample of 2,736 children aged 4-16 years, and their carers, from 1993 until 2007 using administrative record linkage. Children were aged between 18 and 31 years at end of follow-up. Proportional hazards regression was used to examine the relationship between child, parent, family, school and community factors measured in 1993, and subsequent hospitalisation for DSH.

**Results:**

There were six factors measured in 1993 that increased a child's risk of future hospitalisation with DSH: female sex; primary carer being a smoker; being in a step/blended family; having more emotional or behavioural problems than other children; living in a family with inconsistent parenting style; and having a teenage mother. Factors found to be not significant included birth weight, combined carer income, carer's lifetime treatment for a mental health problem, and carer education.

**Conclusions:**

The persistence of carer smoking as an independent risk factor for later DSH, after adjusting for child, carer, family, school and community level socio-ecological factors, adds to the known risk domains for DSH, and invites further investigation into the underlying mechanisms of this relationship. This study has also confirmed the association of five previously known risk factors for DSH.

## Background

A prior episode of deliberate self-harm (DSH) is one of the strongest predictors of future completed suicide [1], therefore identifying antecedents of DSH may inform strategies aimed at reducing suicide rates. Recent extensive reviews of DSH identified similar risk factor domains and conceptual models for self-harm [2-5]. Commonly identified risk factor domains include socio-economic disadvantage, female gender, psychiatric disorders, adverse childhood and family circumstances, and sexual and physical abuse, with the models also reflecting the interlinked nature of these domains in determining risk profiles. Two of these reviews recommended developing more complex and innovative models incorporating greater environmental components and employing longitudinal designs [4,5]. Gratz [4] notes that empirical research has tended to concentrate on the relationship between DSH and childhood abuse and neglect, and suggests future work look to investigate the caregiving relationship and family-related childhood experiences as possible influences on later DSH. Beautrais [5] argues for more longitudinal research on adolescents, with a wider focus than just suicidal behaviour, to better elucidate pathways into the spectrum of problems facing young people.

This study sought to address some of these concerns by utilising a quasi-longitudinal design within a socio-ecological framework, as used in the 1993 Western Australian Child Health Survey (WACHS) [6-8], to identify factors measured in childhood that predict future episodes of DSH. Data collected on 2,736 children aged 4-16 years in the WACHS, a cross sectional survey of health and wellbeing conducted in 1993, were linked to administrative hospital records over the ensuing 14 years until December 2007. At completion of follow-up the original study children were aged between 18 and 31 years. We hypothesised that socio-ecological factors measured earlier in life, in the WACHS, would be predictive of later episodes of DSH identified in linked hospital data over the follow-up period. Other studies have shown DSH to be associated with a range of socio-demographic factors, many of which were available in the WACHS. However, few previous studies have used similar methodology to that used here--linking detailed cross sectional survey data to administrative hospital records over time. One that did use similar methods, by Klomek *et al*, investigated suicide attempts and completed suicides up to the age of 25 years, in relation to detailed bullying information collected at age 8 years, and found differential outcomes by sex [9]. While our study's ability to replicate Klomek *et al's *bullying analysis is beyond the scope of our questionnaire, it was designed to test the association of a wide range of other factors with hospital admissions for DSH.

The DSH cases in our study were serious enough to require hospital admission for treatment, as opposed to treatment in an emergency department only or out-patient clinic only. Therefore these cases likely represent the most severe end of the DSH spectrum, and our sample, interrogated over a 14-year follow-up period and using a reliable hospital record source, contains sufficient DSH cases to allow meaningful relationships to be observed.

## Methods

### Data sources

#### 1993 Western Australian Child Health Survey

This was a face-to-face household survey of 2,736 children and their families in a representative random sample from across Western Australia (WA). The WACHS was predicated on a socio-ecological framework of child development that incorporated child, parent, family, school and community level indicators and measures. The children were aged 4-16 years at the time of interview, and all eligible children in a household were selected. Dwellings were randomly selected and participation in the WACHS was voluntary, with 82% of eligible households agreeing to participate. Survey collection took place from July through September 1993. Personal interview with the primary carer, using trained professional interviewers from the Australian Bureau of Statistics, gathered extensive data from consenting families on demographics, family backgrounds, and children's physical and mental health. Of the primary carers, 97% were the natural mothers of study children, 1.4% were the father, with the remaining 1.6% representing other care arrangements.

Forms were also sent to primary and high schools for each survey child, whereby information on academic performance, temperament and behaviour was gathered from each child's teacher and school Principal. Aboriginal children living in Perth were sampled in proportion to population, which resulted in too low a sample population to allow meaningful analysis. Aboriginal children living outside the Perth metropolitan area were excluded from this study. At the time of WACHS development the same study team were working with Aboriginal groups to design a subsequent child health survey exclusively for Aboriginal children in WA, with tailored questions, an appropriate sampling strategy and sample size. This separate new study went into the field in 2000 [10]. Further details of the 1993 WACHS, including study design, response rates and measures, have been described elsewhere [6-8].

#### Western Australian Data Linkage System (WADLS)

The WADLS is a population database of linked hospital and other health system records. It includes hospital admissions, mortality, midwives, births, cancer, mental health contacts, electoral roll and other related administrative data sets for WA [11]. Information about individuals admitted to hospitals in other Australian states and territories is not available through the WADLS, as these other states and territories represent separate legal jurisdictions and use different recording systems. Jurisdictions other than WA are effectively different geographical and legal catchment areas, which do not presently support routine overlap or on-going cross-jurisdictional data-linkage. The WADLS data used in this study were prepared by the Western Australian Data Linkage Unit (WADLU). WACHS data for children and their carers were linked with health service utilisation data collected between the time of the survey and December 31 2007. Birth records were also obtained for children of the study. The WACHS data custodians provided the list of names and addresses for all 2,736 children and 2,679 carers who participated in the survey to the WADLU for linkage to the WADLS. Using a unique record linkage key, a de-identified, confidentialised file was then sent to the analysts to complete the study.

Of the total 2,736 WACHS children, 2,304 (84%) were born in WA, and therefore 16% did not have a birth record on the WADLS. The oldest WACHS children were born in 1977, but the WADLS contains detailed perinatal records from 1980 onwards. Hence only basic perinatal data for WACHS children born before 1980 were available for this analysis. Also, some children and carers may have left WA since the 1993 WACHS was conducted, meaning that they would not have a WADLS record for any hospital admissions occurring outside of WA. We had no way of reliably assessing how many survey children had moved away from WA for the entire follow-up period or any part thereof. However, as at December 2007 some 86% of the original sample was registered on the WA Electoral Roll as living in WA. Of the 2,304 WACHS children that were born in WA, 2,282 were linked to their birth records (99%). Of the 432 WACHS children that were born outside WA, 355 linked to the morbidity, mental health or electoral roll records (82%).

The Human Research Ethics Committee at Curtin University of Technology approved this data linkage study.

### Classification of deliberate self-harm

DSH was defined by use of relevant codes described in the International Classification of Diseases (ICD). Any admission to a private or public hospital in WA (including psychiatric inpatient admissions) where one or more of these codes was recorded has been identified as an episode of DSH. For cases prior to July 1, 1999 ICD-9-CM [12] was used, codes E950-E959.9: Suicide and self inflicted injury. These codes include: injuries in suicide and attempted suicide; self-inflicted injuries specified as intentional. For cases recorded from July 1, 1999 onwards ICD-10-AM [13] was used, codes X60-X84.99: Intentional self-harm. These codes include: purposely self-inflicted poisoning or injury; suicide (attempted). Fewer than four completed suicides were identified via these codes for this cohort, and these cases were excluded from the analysis presented in this paper to protect the confidentiality of the persons involved.

We also used the following codes to assess each case of harm due to undetermined intent, before excluding them from our analysis on the basis that accident or third party involvement could not be ruled out for each case: ICD-9-CM, codes E980-E989: Injury undetermined whether accidentally or purposely inflicted, and ICD-10-AM, codes Y10-Y34.99: Event of undetermined intent.

### Measures

Reflecting the theoretical basis underpinning the WACHS socio-ecological model, individual child, primary carer, family, school and community level characteristics were examined as potential antecedents of DSH.

Individual child characteristics included the child's sex, an estimate of mental health morbidity using Achenbach's Child Behaviour Checklist (CBCL) [14]--including a combined parent/teacher total CBCL score [15] and eight CBCL syndromes, a general question about their level of emotional and behavioural problems compared with other children their age, intelligence quotient (IQ) measured using British Ability Scales [16], birth weight, gestational age, and whether they were breastfed. Characteristics of the primary carer included whether they were a smoker, maternal age, highest school year completed, the importance of religion in their life, parenting style (four categories: encouraging; coercive; neutral; and inconsistent) [7], self-reported lifetime treatment for emotional or mental health problems up until 1993, hospitalisation with mental health problems and/or DSH since 1993, and whether they held any government benefit cards. In the vast majority of cases, the primary carer of the child was the mother.

Family level characteristics included family type (original, step/blended or sole-parent), combined carer income, and the level of family functioning. Combined carer income, measured in 1993 Australian dollars, was defined as low (less than $600 per week), medium ($600 to $1100 per week) or high (over $1100 per week). The McMaster Family Assessment Device (FAD) was used as a global measure of the health of family functioning [17]. At the school level, academic performance data was collected from each child's classroom teacher at the same time as the household phase of the WACHS. Community level characteristics included whether the family lived in a metropolitan or non-metropolitan area and the Socio-Economic Indexes for Areas (SEIFA). Based on Census information, these SEIFA provide a measure of area 'disadvantage' and can be used to assess socio-economic conditions by geographical areas [18].

### Classification of Mental Health Service Use

Mental health problems resulting in hospitalisation over the follow-up reference period were defined by the following codes from the International Classification of Diseases: ICD-9-CM, Chapter 5: Mental Disorders 290-319, and from July 1, 1999 onwards ICD-10-AM, F00-F99: Mental and behavioural disorders.

### Weighting and estimation procedures

The WACHS was a stratified, clustered representative probability sample. Weights were employed to account for selection probabilities and correct for potential non-response biases, with post-stratification by age, sex, family size and geographic area. Proportions were estimated using the survey weights to produce population-unbiased estimates. We calculated the population weighted proportion of children from the WACHS who had a hospital record for DSH, and then compared these proportions measured against variables from the WACHS. Variances and confidence intervals on estimates were produced using the ultimate cluster variance estimation technique [19]. This accounted for the clustered nature of the original survey sample. Full details of the survey methodology and weighting and estimation procedures have been described elsewhere [6].

All analyses were performed using SAS version 9 except where noted [20].

### Proportional hazards regression

The association between factors collected in the 1993 WACHS and DSH was assessed using multivariate proportional hazards regression. All children in the WACHS were followed for the same length of time, however as they ranged in age between 4 and 16 years in 1993 they have variable risk periods for DSH resulting in hospitalisation. No episodes of hospitalisation with DSH were recorded for children younger than 14 years in this cohort across the follow-up period. As such, for children younger than 14 years at the time of the WACHS, start of follow-up was taken as each child's 14^th ^birthday. For children aged 14 years or older in 1993, start of follow-up was the date of the survey interview. Children were followed to the end of December 2007 or date of first hospital admission for DSH. We included age of child at time of the survey in the model to allow for any possible age-specific cohort effects. The full model using categorical predictor variables was fitted using SAS.

In addition, we fitted a model with maternal age of the child's mother as a continuous variable. As proportional hazards regression models the log of the hazard ratio it is generally not appropriate to assume that the association with a continuous variable will be linear. As there were no theoretical grounds to hypothesise any particular shape for this relationship, we fitted a non-parametric spline curve using generalised additive models. This model was fitted using Hastie and Tibshirani's GAIM software [21].

## Results

There were 46 episodes of DSH resulting in admission to hospital for 37 WACHS children (1.4%) over the follow-up period. The median age of first admission for DSH was 18 years. There were eight cases of injury of undetermined intent. Following an investigation of each case, it was clear that five cases were most likely accidentally inflicted, either by the subject or a third party. Determining intent for the remaining three cases was less conclusive, however the harm recorded was at the lower end of the severity spectrum as no medical procedures were undertaken before same-day hospital discharge for this group. On this basis all eight cases were dropped from the analysis. There were 84 episodes of admission to hospital for DSH by 39 WACHS carers (1.5%) over the follow-up period. There were less than three cases where both a carer and a child who were living in the same household at the time of the 1993 WACHS were later hospitalised for self-harm.

### Associations with DSH among CHS children and other hospital contact for mental disorders

There were 483 hospital in-patient admissions for mental disorders observed for 190 study children. There were 6,306 hospital out-patient episodes for mental disorders observed for 241 study children. Of the study children with service contact for a mental disorder, 99 were treated as both in-patients and out-patients, 91 were treated only as in-patients, and 142 were treated only as out-patients. In total, 332 children had service contact for mental disorders (12.1%).

Of the 37 study children who presented at hospital with an episode of DSH, seven (19%) had also been diagnosed with a mental disorder in the WADLS prior to their first DSH admission.

### Population weighted bivariate analysis

Table 1 reports the population weighted proportions of children from the WACHS who went on to be hospitalised for DSH, by a range of variables that were part of the WACHS socio-ecological model of child development [6-8].

**Table 1:** Population weighted proportions of WACHS children who were hospitalised with at least one episode of deliberate self-harm between interview in 1993 and December 31 2007, by selected items from the WACHS

	Hospitalised for DSH (n = 37)	Not hospitalised for DSH (n = 2,699)
	Estimate (95% CI)	Estimate (95% CI)
*Child level factors*		
Sex		
--Male	30.3% (14.3%-51.8%)	50.1% (48.0%-52.3%)
--Female	69.7% (48.2%-85.7%)	49.9% (47.7%-52.0%)
Emotional problems		
-No emotional problems	53.8% (35.7%-73.6%)*	79.7% (77.7%-81.6%)*
-Emotional problems NOT more than other children	9.1% (1.7%-21.9%)	8.4% (7.3%-9.7%)
-Emotional problems MORE than other children	37.0% (18.8%-59.4%)*	10.0% (8.6%-11.6%)*
CBCL Total Score:		
-Normal	63.3% (43.9%-80.1%)	79.0% (77.1%-80.8%)
-Abnormal	28.6% (13.7%-46.7%)	16.7% (15.0%-18.5%)
CBCL: Delinquent Behavior Score:		
-Normal	64.3% (43.9%-80.1%)	86.8% (85.2%-88.3%)
-Abnormal	27.6% (13.7%-46.7%)*	8.9% (7.7%-10.2%)*
Ever breastfed		
-No	12.7% (4.1%-26.2%)	15.9% (13.8%-18.2%)
-Yes	87.3% (73.8%-95.9%)	83.8% (81.5%-85.9%)
Birth weight		
-Under 2500 g	0.0% (0.0%-1.3%)	3.5% (2.7%-4.4%)
-2500 g and over	71.3% (49.8%-86.2%)	59.4% (56.6%-62.3%)
-Not known	28.7% (13.8%-50.2%)	37.1% (34.3%-39.9%)
IQ score 1993		
51-79	6.3% (1.4%-18.3%)	8.8% (7.6%-10.3%)
80-119	45.8% (26.4%-64.3%)	51.6% (49.1%-54.1%)
120-149	4.8% (1.1%-14.6%)	9.8% (8.4%-11.4%)
*Carer level factors*		
Carer smoking status		
-Non-smoker	48.0% (26.6%-66.6%)*	74.6% (71.5%-77.5%)*
-Current smoker	52.0% (33.4%-73.4%)*	24.8% (21.9%-27.9%)*
Highest school year completed by child's carer		
-Year 9 or lower	16.0% (3.6%-41.4%)	13.2% (11.1%-15.6%)
-Year 10 or higher	84.0% (60.4%-96.6%)	85.8% (83.3%-88.0%)
Government benefit card status of child's carer		
-No benefit card	53.8% (36.0%-72.7%)	62.9% (59.3%-66.5%)
-Holds benefit card	46.2% (27.3%-64.0%)	36.5% (32.9%-40.0%)
Carer reported lifetime treatment for mental health problems as at 1993		
-Yes, have been treated	29.3% (12.7%-47.2%)	11.4% (9.6%-13.3%)
-No, never treated	70.7% (52.8%-87.3%)	87.9% (85.9%-89.8%)
Parenting style		
-Encouraging	23.4% (11.8%-41.2%)*	49.4% (46.9%-52.0%)*
-Coercive	6.8% (1.3%-17.2%)	5.1% (4.2%-6.1%)
-Neutral	16.0% (3.0%-36.3%)	7.0% (5.8%-8.3%)
-Inconsistent	53.8% (34.7%-70.9%)	38.2% (35.6%-40.8%)
Importance of religion to carer		
-Very important	16.1% (7.0%-31.4%)	20.8% (18.2%-23.6%)
-Reasonably important	52.9% (33.1%-69.8%)	33.6% (30.8%-36.4%
-Not important at all	22.9% (7.1%-42.2%)	40.0% (36.7%-43.4%)
Maternal age at birth		
- < 20 years	27.5% (11.6%-47.8%	5.6% (4.2%-7.1%)*
- > = 20 years	72.5% (52.2%-88.4%)*	93.4% (91.7%-94.8%)*
*Family level factors*		
Family type		
-Original	46.2% (25.5%-64.7%	74.2% (70.9%-77.2%)*
-Step/blended	28.8% (10.7%-50.2%)	9.3% (7.7%-11.1%)
-Sole parent	25.0% (9.1%-51.2%)	16.5% (13.8%-19.4%)
Combined weekly income of child's carers (1993 Australian dollars)		
-Low (<$600)	42.3% (26.4%-62.3%)	38.8% (35.2%-42.7%)
-Medium ($600-$1,100)	34.8% (19.7%-53.5%)	38.9% (35.6%-42.4%)
-High (>$1,100)	16.1% (6.4%-32.8%)	14.8% (12.3%-17.5%)
Family functioning (FAD)		
-Good	87.1% (66.9%-98.7%)	86.5% (84.2%-88.5%)
-Poor	0.5% (0.0%-3.8%)	0.3% (0.1%-0.7%)
*School level factors*		
Teacher rated academic performance at school		
-Far below age	4.2% (0.6%-15.8%)	2.1% (1.5%-2.8%)
-Somewhat below age	5.7% (0.6%-16.5%)	11.8% (10.3%-13.6%)
-At age level	37.7% (18.0%-57.5%)	33.4% (31.3%-35.7%)
-Somewhat above age	12.9% (4.4%-28.1%)	19.4% (17.5%-21.5%)
-Far above age	0.0% (0.0%-1.3%)	4.3% (3.4%-5.4%)
*Community level factors*		
Metropolitan or rural residence		
-Metro	71.4% (50.6%-85.3%)	69.3% (63.9%-74.2%)
-Rural	20.5% (10.5%-35.0%)	26.3% (22.4%-30.4%)
SEIFA index of relative socio-economic disadvantage		
-Less than 950 (most disadvantaged)	31.9% (14.3%-51.8%)	20.3% (14.3%-26.8%)
950-1000	16.8% (7.0%-35.5%)	19.4% (13.7%-26.4%)
1000-1060	25.7% (9.8%-46.7%)	30.0% (22.7%-38.3%)
Over 1060 (least disadvantaged)	25.6% (10.7%-50.2%)	30.4% (22.9%-38.0%)

* = Significant at 95% confidence level.

#### Child factors

More than twice the proportion of females were hospitalised for DSH, compared with males. This did not quite reach statistical significance. For children who were later hospitalised for DSH, 53.8% were said by their carers to have 'no emotional or behavioural problems' in the six months prior to the survey, whereas among those not hospitalised with DSH 79.7% were reported to have no emotional problems. Similarly, 37.0% of children who went on to be hospitalised with DSH were said by their carers to have 'more emotional or behavioural problems' than other children their age, compared with 10.0% of those children not hospitalised for DSH. Of those children who were later hospitalised with DSH, 27.6% were rated in the "Abnormal" range on the CBCL Delinquent Behaviour syndrome scale, compared with 8.9% of those with no record of self-harm. No significant outcome was observed for the other seven CBCL syndromes, nor for the CBCL total score.

#### Carer factors

Some 52.0% of children hospitalised for DSH had a primary carer who was a current smoker in 1993, compared with 24.8% of children who did not present with self-harm. Of those children hospitalised for DSH, 27.5% were born to a teenage mother, compared with 5.6% of children who did not present with self-harm. Less than one-quarter (23.4%) of children hospitalised with DSH lived in a household where the parenting style was 'encouraging' in 1993, compared with almost half (49.4%) those children not hospitalised with DSH. There were no significant differences for the other three categories of parenting style. However, 'inconsistent' parenting style did approach significance, with 53.8% of children hospitalised with DSH recording 'inconsistent' parenting style in 1993, against 38.2% for those children with no DSH record.

#### Family factors

Of children hospitalised with DSH, 46.2% were living in a two-parent original family at the time of the WACHS. In contrast, of children not hospitalised for DSH, 74.2% were living in original families in 1993. No significant difference was observed with step/blended or sole-parent families.

Other factors in our socio-ecological model were examined for bivariate associations with later hospitalisation for DSH and found to be non-significant. These included--*Child factors*: Combined parent/teacher CBCL total score; Whether child was breastfed as an infant; Whether child was classified as a low birth weight baby (under 2,500 g); Child's IQ score in 1993. *Carer factors*: Highest school year completed by child's primary carer; Government benefit card status of child's primary carer; Carer reported lifetime treatment for mental health problems as at 1993; Importance of religion to child's primary carer in 1993. *Family factors*: Combined weekly income of child's carers; Family functioning. *School factors*: Teacher rated academic performance at school. *Community factors*: SEIFA index of relative disadvantage; metropolitan versus rural residence in 1993.

### Proportional Hazards Model

A proportional hazards model was built to investigate which factors from the WACHS socio-ecological model of child development were independent predictors of increased risk for future hospitalisation with DSH. All variables used in the bivariate analyses were tested in the process of obtaining the most parsimonious set of DSH risk factors.

Table 2 shows multivariate hazard ratios of modelled predictors of hospitalisation for DSH over the 14-year follow-up period for WACHS children. *Child factors*: Females were at 3.53 times the risk of males to be hospitalised for DSH. There was no significant difference in DSH hospitalisation by age group, which suggests there was no age-cohort effect in DSH among the study children. Children reported by their carers at the time of the survey to have 'more emotional or behavioural problems' than other children their age were at 3.47 times the risk for subsequent hospitalisation with DSH than children reported to have no emotional or behavioural problems. *Carer factors*: Children whose primary carer was a current smoker in 1993 were at 3.02 times the risk for hospitalisation with DSH than children whose primary carer was a non-smoker. Compared with children living in households in 1993 where parenting style was classified as 'encouraging', children living in households where parenting style was classified as 'inconsistent' were at 2.31 times the risk for hospitalisation with DSH. No significant difference was observed for either 'coercive' or 'neutral' parenting styles, although the risks were elevated for both. Children born to a teenage mother were at 2.70 times the risk for hospitalisation with DSH than children born to a mother aged 20 years or older. *Family factors*: Children living in a step/blended family arrangement in 1993 were at 2.28 times the risk for hospitalisation with DSH than children in two-parent original families. No significant difference was observed for children living in sole-parent families.

**Table 2:** Multivariate hazard ratios for hospitalisation with deliberate self-harm over a 14 year follow-up period, for children aged 4-16 years in 1993.

	Hazard Ratio	95% CI
Factor		
Sex		
Female vs. Male	3.53***	1.69-7.38
Age group (years)		
12-16 vs. 4-11	1.22	0.57-2.60
Primary carer smokes		
Yes vs. No	3.02**	1.53-5.95
Family type		
Sole parent vs. original	1.08	0.46-2.54
Step/blended vs. original	2.28*	1.01-5.15
Emotional problems		
NOT more than other children vs. None	0.94	0.27-3.24
MORE than other children vs. None	3.47**	1.65-7.31
Parenting Style		
Coercive vs. Encouraging	2.53	0.69-9.29
Inconsistent vs. Encouraging	2.31*	1.03-5.18
Neutral vs. Encouraging	2.79	0.88-8.88
Maternal age at birth		
Mother aged < 20 years vs. > = 20 years	2.70*	1.20-6.06

*p < .05; **p < .01;***p < .001.

#### Items eliminated from the final model

No school or community level factors were found to be significant in the final model. Individual variables that were eliminated in the process of obtaining the most parsimonious model included: prior use of mental health services by the child or the carer; CBCL total score and subscales; household income; benefit card status; carer education; SEIFA; birth weight; gestational age; breastfeeding status; child's IQ; and child's academic performance in school.

### Maternal age and deliberate self-harm

**Figure 1:**
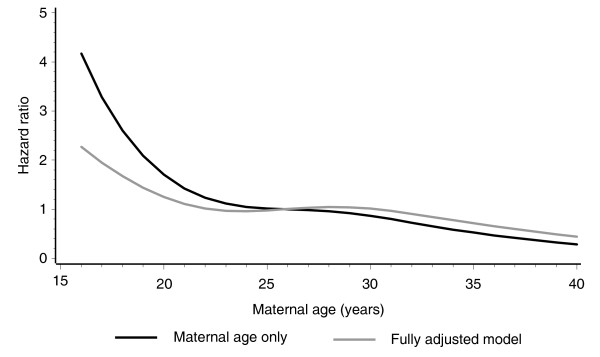
**Unadjusted and adjusted hazard ratios for hospitalisation with deliberate self-harm over a 14-year follow-up period, for children aged 4-16 years in 1993, by maternal age of child's carer**.

In order to investigate the shape of the relationship between DSH and maternal age we used non-parametric spline modelling. Two models were fitted, one with maternal age only, and another including maternal age and adjusting for all items from the proportional hazards model shown in Table 2. These are depicted in Figure 1, which shows that hazards for DSH rise sharply with decreasing maternal age in the teenage years, both with maternal age as an unadjusted variable and also when adjusted for confounding by the other variables from the proportional hazards model.

## Discussion

At the outset we sought to expand the empirical scope of existing DSH research by utilising a socio-ecological framework represented by the 1993 WACHS in a quasi-longitudinal study design through data-linkage to the health system. This methodology also allowed us to test multi-generational influences on DSH. Individual, primary carer, family, school and community level characteristics were examined as potential predictors of DSH. These data support our hypothesis that socio-ecological factors measured in children aged 4-16 years are predictive of later episodes of hospital recorded DSH over a 14-year follow-up period. Results of this study identified one new risk factor that predicts later episodes of DSH--carer smoking-- and confirmed several others already known in the literature.

Deliberate self-harm is a term that has been used in the literature to describe actions intended to inflict pain, harm, disfigurement, or in extreme cases, death (but not actually resulting in death), on one's self. Clearly these actions may span a wide spectrum of severity and risk for completed suicide. There is ongoing debate among researchers as to what the term "deliberate self-harm" actually encompasses, and whether the term should include cases of attempted suicide along with self-harm cases with no intent to suicide [4,22]. This paper does not inform that debate, as hospital records used for this study do not distinguish between people who intended non-fatal harm from those whose intent was suicide. As we cannot state with certainty that all cases were suicide attempts, regardless of the severity of their self-inflicted injuries, we have used the term "deliberate self-harm" in preference to "attempted suicide" to refer to actions resulting in hospitalisation for the cases here. Whilst not wishing to add to what Linehan [23] described as "definitional obfuscation" around various suicidal behaviours, and non-suicidal but still self-harming behaviours, we needed to use one of the recognised terms to represent our cases, and have chosen the term that we feel is least misleading for our study. Regardless of fatal intent, people who have previously self-harmed remain at higher risk for suicide attempt and completed suicide [1,24,25].

Beginning with child factors, we identified two independent predictors of future DSH operating at this level. Female children were at higher risk than male children for hospital admission with DSH. Higher incidence of DSH among females is well established in the literature [26-28]. Children who had more emotional and behavioural problems than other children their age, as reported by their primary carer in 1993, were at increased risk for hospitalisation with DSH later in life. Mental health problems are known to be associated with instances of DSH among individuals [29,30]. Early identification of emotional and behavioural problems could assist with targeting of counselling and treatment services, which in turn could mitigate later episodes of DSH.

We found no relationship between birth weight, or proportion of optimal birth weight, and hospitalisation for DSH later in life. Other research using the 1993 WACHS identified a relationship between percentage of expected birth weight and CBCL total score [31]. At least one other study has shown a relationship between DSH and birth weight [32].

Experience of sexual abuse during childhood has been shown to be associated with suicidal behaviour in many other studies [27,33,34]. We were unable to test for this association as a reliable measure of sexual abuse was not available.

Three carer factors were identified as independent risk factors for future DSH. Children born to a teenage mother were at higher risk for hospitalisation with DSH later in life. This finding is supported by others [32,35]. There may be factors associated with becoming a teenage mother, such as socio-economic disadvantage, unstable home environments, and the stress that often accompanies such circumstances, which contribute to future mental health problems in their children. Our study included no data on the mother's general life circumstances leading up to her pregnancy and the intervening period between birth of the study child and the time of the survey, which limited us from investigating the relationship further.

Parenting practices may also be associated with increased risks of subsequent hospitalisation for DSH. Relative to an 'encouraging' parenting style, all other parenting styles showed an elevated risk of subsequent DSH with 'inconsistent' parenting reaching statistical significance. There are established associations in the literature between parenting styles and higher risks of social and emotional problems [7].

Unexpectedly, we have found cigarette smoking by the child's primary carer to be an independent predictor of later DSH by the child. Carer smoking remained significant despite adjustment for a wide range of demographic and psycho-social variables that might otherwise have confounded the association. One variable that may have influenced this result was the mental health status of carers. We had access to hospital records of carers from 1993 onwards, but only a minority of people with mental health problems seek or receive treatment for them in a hospital setting [36]. We also had carer reported data on lifetime treatment for mental health problems from the 1993 WACHS. However, including both of these variables in the model had no effect on the level of risk attributed to carer smoking. A comprehensive measure of parental mental health was not available in this study. There is an established positive association between smoking and mental health problems [37,38], and children of parents with mental health problems are more likely to develop mental health problems themselves [39]. We also had no way of knowing whether any of the study children hospitalised for DSH were current smokers at the time of their hospital admission. As tobacco smoking is known to be associated with attempted suicide [40-45], and children of smokers are more likely to be smokers themselves [46], it is possible that these factors have also contributed to our finding that carer smoking is associated with later admission for DSH by the child. The relationship between mental health and smoking should be investigated further to elucidate the role of smoking by carers in future episodes of DSH by their children.

No relationship was found between children who were hospitalised for DSH later in life and carers who were hospitalised for either DSH or mental health problems over the same period. As child and parental mental health problems are related [39], we investigated the relationship between carer hospitalisation for mental health problems and child self-harm, and found no association. While we were able to test for prior use of mental health services by carers, not all people with mental health problems obtain treatment for their condition in the hospital system, and many go untreated and/or undiagnosed altogether.

One family level factor was found to be associated with future hospitalisation for DSH. Children who were living in a step or blended family arrangement in 1993, compared with those living in original two-parent families, were at elevated risk for hospitalisation for DSH later in life. It was not possible from our data to determine the contribution of the break-up of the original family, the circumstances of the new step/blended relationship, or the combination of these two issues, to later episodes of DSH. We can only state that, in a model adjusted for the child's age-group, children living in step or blended families in 1993 were at higher risk for hospitalisation with DSH than children in original two-parent or sole-parent families. Other studies have demonstrated that dissolution of the parental relationship can increase the risk for suicide attempt [27,47], but few have looked at the differential effect of step/blended and sole-parent family structures. An investigation by Garnefski and Diekstra supported this finding that children in step-parent families are at higher risk for suicide attempt [48].

We found no relationship between later hospitalisation of children for DSH, and previous hospitalisation for mental disorders. A relationship between psychiatric disorders and DSH has been shown elsewhere [29,30], so perhaps a therapeutic benefit accrues from being treated for a mental disorder. The 1993 WACHS showed prevalence of mental health problems among WA children (18%) was much higher than the treatment rate (2%) over the 6 months prior to the study [6]. It could be that those children who self-harm do have mental health problems in the period before their presentation with DSH, but go undiagnosed and untreated, contributing to our finding no relationship between prior hospitalisation for mental disorder and self-harm.

### Strengths and limitations

A key strength of this study was the methodology. Follow-up via data-linkage to administrative datasets conferred several advantages over a traditional longitudinal follow-up, such as: being far more cost effective than face-to-face follow-up due to minimal search costs; permission to link was granted by an ethics committee, eliminating participant loss due to consent bias; reduced respondent bias as there is less risk of general loss to follow-up; and, no reliance on respondent memory or bias in answering questions about sensitive personal issues across a long time period. Hospital data provided a reliable record of serious DSH over time, and the WACHS provided a range of possible antecedents within a socio-ecological framework. Several articles have been published which support the efficiency of this methodology using the WADLS as an example [11,49].

This study used hospital admission data only to identify self-harm cases, as opposed to emergency department, out-patient clinic, general practitioner, or any other medical service usage data. Due to this methodological issue, cases in our study likely fall at the severe end of the DSH spectrum. Whilst hospital admissions data was of high quality, the hospital emergency data was inadequate to allow analysis with regard to either DSH or mental health presentations. Additionally, records of treatment by general practitioners, or of private psychiatrists or psychologists seeing patients in their consulting rooms outside the hospital system were not available to us. To what extent DSH or mental health disorders were treated in these settings we are unable to speculate. As well, it is reasonable to assume that some people who self-harmed, and perhaps more people with mental health disorders, never sought treatment for their condition from either hospital services or private practitioners. It is possible that risk factors associated with DSH serious enough to require hospitalisation may differ from risk factors associated with less serious DSH. It is also possible that some genuine suicide attempts may not result in hospital admission, due to a lower level of harm being inflicted, or treatment occurring in another setting. Our study is unable to investigate these issues. It is impossible to know the true rate of DSH, and the distribution of severity, among our study sample or in the general population. However, logic suggests that serious cases of DSH, many of which might be life threatening regardless of intent, would be more likely to result in hospital admission.

Social attitudes to smoking may have changed during the follow-up period. Certainly in Australia, smoking rates have been reducing steadily since the 1970s [50]--a period when many of the WACHS carers who were current smokers at the time of the survey would have taken-up the habit--and the social characteristics of persons taking-up smoking in the current era may be different compared with past eras when smoking was more socially mainstream. As smoking rates fall in the mainstream, research shows those continuing to smoke, and those beginning the habit, are more likely to suffer from mental health problems than non-smokers [51,52]. A recent paper has suggested a role for secondhand smoke in the development of psychological distress and future psychiatric illness in healthy adults [53]. These observations suggest the link we have observed between DSH and carer smoking may appear stronger if a similar study to the 1993 WADLS were run today.

## Conclusions

This study confirms several known risk domains for DSH, and identifies carer smoking as an independent risk factor for DSH after adjusting for child, carer, family, school and community level socio-ecological variables. Further research is needed to elucidate the underlying mechanisms of the relationship between carer smoking and DSH.

## Competing interests

The authors declare that they have no competing interests.

## Authors' contributions

SZ, SS and FJS conceived the original idea for this data linkage study. All authors contributed to the development of the study methodology. FM undertook the data analysis and wrote the first draft of the manuscript, with assistance from DL and JG. All authors edited the paper. All authors read and approved the final manuscript.

## Pre-publication history

The pre-publication history for this paper can be accessed here:

http://www.biomedcentral.com/1471-244X/10/82/prepub
